# Correction: Contemporary trends of witchcraft accusations and resulting violence against children: A scoping review and bibliometric analysis protocol

**DOI:** 10.1371/journal.pone.0348210

**Published:** 2026-04-27

**Authors:** Cara Spence, Edward Salifu Mahama, Kimberly Jarvis, Mary Zettl, Vida Nyagre Yakong, Mary Ani-Amponsah, Helen Vallianatos, Samuel Adjorlolo, Courage Kosi Setsoafia Saba, Michael Frishkopf, Geoffrey Maina, Solina Richter, Pammla Petrucka

Michael Frishkopf is not included in the author byline. Michael Frishkopf should be listed as the tenth author and affiliated with University of Alberta, Canada. The contributions of this author are as follows: Conceptualized research goals and aims.

The ninth author’s name is spelled incorrectly. The correct name is: Courage Kosi Setsoafia Saba.
