# Supplementary material for: Contemporary trends of witchcraft accusations and resulting violence against children: A scoping review and bibliometric analysis protocol
Source: PLoS One. 2026 Feb 2;21(2):e0338997. doi: 10.1371/journal.pone.0338997 (PMC12863508; doi:10.1371/journal.pone.0338997)
Supplement: S2 File — (DOCX) [file pone.0338997.s002.docx]

# SI 2: PCC Framework for JBI Scoping Review

| **Topic: Witchcraft-related violence against children across the globe** | | | | |
| --- | --- | --- | --- | --- |
| **Objective(s):** This review seeks to understand the global trends of contemporary witchcraft accusations and related harms against children and adolescents (0-18 years of age). | | | | |
| **Primary review question:** To what extent does harm or violence against children and adolescents (ages 0-18) result from witchcraft-related accusations in the global context? | | | | |
| **Secondary Question(s):**1) What is the prevalence of perceptions, beliefs, and lived experiences of “witchcraft” accusations and actions against children and adolescents (ages 0-18)?a) What was the specific accusation made against the victim(s)?b) Under what circumstances are accusations made?c) Who were the accusers, and what were the relationships with the accused?d) What were the resulting outcomes of the accusation? (e.g. type(s) of harm)e) What are the lasting impacts on the accused (related to the accusation, and theresulting harm?2) How do accusations of “witchcraft” impact children and adolescents, specifically in terms of social welfare, well-being, safety, gender-based violence, and socio-economic vulnerability?3) What historical legacies, customary laws, tribal practices, public discourse, and social and religious beliefs support allegations and facilitate witchcraft-related harms within the country or region?4) What tools, resources, and approaches have been implemented to address the harmful impacts and outcomes of witchcraft practices and allegations against children and adolescents globally? | | | | |
| **PCC Element** | **Definition (per JBI Reviewer’s Manual Ch.11)** | | **Project Scope** | |
| Population | *“Important characteristics of participants, including age and other qualifying criteria” (11.2.4)*  You may not need to include this element unless your question focuses on a specific condition or cohort. | | Children/ adolescents: ages 0-15  Adolescents ages: 16-18 will be excluded as they are generally grouped in with adults in many places in the world. They may be included in this review if search findings present or include these ages in their definition of ‘children’ or ‘adolescent’ | |
| **Concept** | *“The core concept examined by the scoping review should be clearly articulated to guide the scope and breadth of the inquiry. This may include details that pertain to elements that would be detailed in a standard systematic review, such as the “interventions” and/or “phenomena of interest” and/or “outcomes” (11.2.4)* | | - Witchcraft accusations against children and adolescents ages 0-18 - Actions taken resulting from such accusations of witchcraft - Impacts resulting from actions taken against these children accused of witchcraft - Interventions aimed at addressing and reducing witchcraft accusations and related actions against children - Interventions aimed at supporting those children who experienced violence or who are vulnerable to violence as a result of witchcraft accusations | |
| **Context** | “*May include… cultural factors such as geographic location and/or specific racial or gender-based interests. In some cases, context may also encompass details about the specific setting.”* | | - Globally - All settings especially within home, within communities, communal spaces (i.e. markets, houses of worship/prayer), family, at school - All sexes and genders - All sexual orientations - Any faith or religious belief system | |
| **Other:** | **Criteria** | | **Justification** | |
| **Geographical focus** | Globally;  Sub-Saharan Africa (priority) | | A 2013 report by the EU, highlighted that witchcraft accusations against children are on the rise not only in sub-Saharan Africa but also Bolivia, Guatemala, Haiti, India, Indonesia, Islamic Republic of Iran, Mexico, Nepal, Pakistan, Papua New Guinea, Thailand, Saudi Arabia and Syria. (Hanson & Ruggiero, 2013)  As such, this review will look globally across all continents but with specific interest in Sub-Saharan Africa. | |
| **Language(s)** | ALL | | English is the primary publication language but much of the grey literature available may be in a wide range of local languages due to the prevalence of beliefs across a host of countries. To ensure a sufficiently wide/comprehensive search has been carried out, all languages will be included in the initial search results. Google translate will be used to help review articles for eligibility with the inclusion criteria. | |
| **Year range** | 1946 - 2025 | | As this review is the first of its kind that the team knows of, a long historical range is warranted. | |
| **Document type(s)** | Scientific/academic journals  Reports  Newspaper articles  Policies  Theses  Working papers | | cross-cultural, descriptive observational study designs, systematic reviews, case series, prospective and retrospective cohort studies, reports, case studies, individual case reports, and descriptive cross-sectional studies extracted from relevant academic literature databases and considered for inclusion. Study designs include phenomenology, grounded theory, ethnography, qualitative description, action research, social work theory, and feminist research. Text, opinion papers, official reports, thesis, and ‘grey literature’ will be extracted from a worldwide Google web search and grey literature databases to be considered for inclusion. | |
| **Databases** | • Academic Search Complete  • Global Health  • MEDLINER  • Public Health  • PubMed  • Social Services Abstracts  • ProQuest  • JSTOR  • Taylor & Francis  • Emerald  • OARE  • Sage Journals  • Springer  • Wiley  • Project Muse  • Ingenta Connect  • Eric  • eHRAF  • SCOPUS  • Gale Academic One  • Ovid Medline  • Embase (via embase.com)  • CINAHL Plus (via EBSCOhost)  • APA PsycInfo (via EBSCOhost)  • Anthropology Plus (via EBSCOhost)  • ERIC (via EBSCOhost)  • SocINDEX (via EBSCOhost)  • Women's Studies International (via EBSCOhost)  • International bibliography of the social sciences (via ProQuest) | |  | |
| **Grey Literature databases/ search engines** | • ProQuest Dissertations and Theses  • OATD: Open Access Theses and Dissertations  • Bielefeld Academic Search Engine  • OAIster  • OpenGrey  • Grey Source Index  • Grey Guide  • Google  • UGSpace  • African Journals Online  • African Index Medicus (AIM)  • ELDIS   - Middle Eastern and North African Newspaper | |  | |
| **Study Designs** | Qualitative, quantitative, mixed methods, program evaluations, quality improvement reports, organizational reports, annual reports, dissertations, theses, peer reviewed conference papers, opinion pieces will all be considered for inclusion | | | |
| **Exclusion criteria** | - Any publication that does not outline or include specific details of witchcraft-related accusations and related harm against children/adolescents. (Do not meet the eligibility criteria outlined above). - Publications that discuss witchcraft, spiritual or magic beliefs in the context of healing or related to religions or worldviews. - Publications focused on magical or spiritual beliefs of children. - Publications that focus on individuals labelled as ‘magical’ or ‘spiritual’ following a medical procedure or illness. - Any publication that has a different application of one of the search terms than that related to witchcraft accusations and related harm against individuals ages 0-18 (e.g. fetish – used in Africa in relation to witchcraft; but also used to refer to a sexual predilection.). - Any publication that does not report on the harm incurred on the child as a result of the respective accusation, ritual or belief. | | | |
| **Duplicate screener and remover** | Covidence – screening software for reviewing citations for deduplication; reviewing abstracts, resolving disputes among team members | | | |
| **Search Terms** | **Concept** | **Keywords** | | **Subject terms** |
|  | **Witchcraft** | Witch* OR voodoo OR juju OR fetish OR wizard* OR Sorcer* OR “black magic” OR “spiritual practices” OR supernatural OR exorcism OR Curses OR  Magico-religious OR “spiritual possession” OR demons OR “faith healing” OR satan* OR Occulti* OR  “spiritual warfare”      (magic not ("Mesenteric Growth Improves Circulation" or "magic trial")).af.    (evil OR malevolent) AND Spirits | | spiritual therapies/ or faith healing/ or magic/ or medicine, african traditional/ or shamanism/ or witchcraft/ or  exp Occultism/ or superstitions/    **APA psycINFO terms -**  superstitions/ or taboos/ or exp faith healing/ or spirit possession/ or  witchcraft/ or faith healing/ or mysticism/ or occultism/ or shamanism/ |
|  | **children or adolescent** | Child* OR teen* OR toddler OR infant OR adolescent OR Pre-teen OR youth OR baby OR pediatric* | | adolescent/ or child/ or child, preschool/ or infant/ or infant, newborn/ |
|  | **accusation** | Accusation OR claim OR charge OR indictment OR assertion OR blame OR allegation | |  |
|  | **ritual** | belief or practice or ritual* or custom | | Ceremonial Behavior/ |
