# Supplementary material for: Contemporary trends of witchcraft accusations and resulting violence against children: A scoping review and bibliometric analysis protocol
Source: PLoS One. 2026 Feb 2;21(2):e0338997. doi: 10.1371/journal.pone.0338997 (PMC12863508; doi:10.1371/journal.pone.0338997)
Supplement: S3 File — (DOCX) [file pone.0338997.s003.docx]

## SI 3: Literature Search Strategy

Below outlines the search strings developed with a library scientist and the preliminary results across a test of five targeted databases.

### Academic Publications

| **Database** | **Search Terms** | **Results** |
| --- | --- | --- |
| Sociological Abstracts | summary(Witch* OR magic OR voodoo OR juju OR fetish OR wizard* OR Sorcer* OR "black magic" OR "spiritual practices" OR supernatural OR exorcism OR Curses OR Magico-religious OR "spiritual possession" OR demons OR "faith healing" OR satan* OR Occult* OR "spiritual warfare") AND summary(Child* OR teen* OR toddler OR infant OR adolescent OR Pre-teen OR youth) AND summary(Accusation OR claim OR charge OR belief OR practice PR ritual OR indictment OR assertion OR blame OR allegation) | 396 |
| Medline |  | 495 |
| Global Health |  | 60 |
| APA PsycINFO |  | 635 |
| EMBASE |  | 413 |

### Grey literature and Internet

| **Search Engine** | **Search String** | **Results** |
| --- | --- | --- |
| Google & Google Scholar | (witchcraft OR magic OR voodoo OR “faith healing” OR “spiritual warfare”) AND (children OR teens OR youth OR baby OR infant OR child) AND (Accusation OR claim OR charge OR indictment OR assertion OR blame OR allegation OR belief OR practice OR ritual OR custom) | 3.7 million  Will review and hand search first 20 pages. |
