# Supplementary material for: Contemporary trends of witchcraft accusations and resulting violence against children: A scoping review and bibliometric analysis protocol
Source: PLoS One. 2026 Feb 2;21(2):e0338997. doi: 10.1371/journal.pone.0338997 (PMC12863508; doi:10.1371/journal.pone.0338997)
Supplement: S4 File — (DOCX) [file pone.0338997.s004.docx]

| Appendix S4. Data Extraction Tool – Adapted from JBI template source of evidence details, characteristics and results extraction instrument. (Original created: March 26, 2024; Adapted for this study: June 10, 2025) | |
| --- | --- |
| **Scoping Review Details** | |
| **Scoping Review title:** | Contemporary trends of witchcraft accusations and resulting violence against children: A Scoping Review and Bibliometric Analysis Protocol |
| **Review objective/s:** | This review seeks to understand the global trends of contemporary witchcraft accusations and related harms against children and adolescents (0-18 years of age). |
| **Review question/s:** | Primary: To what extent does harm or violence against children and adolescents (ages 0-18) result from witchcraft-related accusations in the global context?  Secondary: 1) What is the prevalence of perceptions, beliefs, and lived experiences of “witchcraft” accusations and actions against children and adolescents (ages 0-18)?a) What was the specific accusation made against the victim(s)?b) Under what circumstances are accusations made?c) Who were the accusers, and what were the relationships with the accused?d) What were the resulting outcomes of the accusation? (e.g. type(s) of harm)e) What are the lasting impacts on the accused (related to the accusation, and theresulting harm?2) How do accusations of “witchcraft” impact children and adolescents, specifically in terms of social welfare, well-being, safety, gender-based violence, and socio-economic vulnerability?3) What historical legacies, customary laws, tribal practices, public discourse, and social and religious beliefs support allegations and facilitate witchcraft-related harms within the country or region? 4) What tools, resources, and approaches have been implemented to address the harmful impacts and outcomes of witchcraft practices and allegations against children and adolescents globally? |
| **Inclusion/Exclusion Criteria** | |
| **Population** | Children/ adolescents: ages 0-15  Adolescents ages: 16-18 will be excluded as they are generally grouped in with adults in many places in the world. They may be included in this review if search findings present or include these ages in their definition of ‘children’ or ‘adolescent’ |
| **Concept** | • Witchcraft accusations against children and adolescents ages 0-18  • Actions taken resulting from such accusations of witchcraft  • Impacts resulting from actions taken against these children accused of witchcraft  • Interventions aimed at addressing and reducing witchcraft accusations and related actions against children  • Interventions aimed at supporting those children who experienced violence or who are vulnerable to violence as a result of witchcraft accusations |
| **Context** | • Globally  • All settings especially within home, within communities, communal spaces (i.e. markets, houses of worship/prayer), family, at school  • All sexes and genders  • All sexual orientations  • Any faith or religious belief system |
| **Types of evidence source** | Scientific/academic journals  Reports  Newspaper articles  Policies  Theses  Working papers |
| **Evidence source: Details and Characteristics** | |
| **Citation details**  (e.g. author/s, date, title, journal, volume, issue, pages) |  |
| **Country** |  |
| **Publication Language** |  |
| **Context** |  |
| **Participants** (details e.g. age/sex and number) |  |
| **Details/Results extracted from source of evidence**  (in relation to the concept of the scoping review) | |
| Specific accusation(s). |  |
| Circumstances under which the accusation(s) were made. |  |
| Identity of accuser(s) (E.g. age, sex, occupation, role in community, etc.) |  |
| Relationship of accuser to victim. |  |
| Outcome(s) of accusation and resulting harm. |  |
| Primary harm (direct). |  |
| Secondary harms (indirect). |  |
| Lasting impacts on the accused/victim(s). |  |
| Consequence (if any) for accuser. |  |
| Support or interventions provided to victim(s). |  |
| Resources or tools cited to support victim(s). |  |
| Policies/laws related to this topic. |  |
